# Online Health Check for Reducing Alcohol Intake among Employees: A Feasibility Study in Six Workplaces across England

**DOI:** 10.1371/journal.pone.0121174

**Published:** 2015-03-23

**Authors:** Zarnie Khadjesari, Dorothy Newbury-Birch, Elizabeth Murray, Don Shenker, Louise Marston, Eileen Kaner

**Affiliations:** 1 Department of Primary Care and Population Health, University College London, London, United Kingdom; 2 Institute of Health & Society, Newcastle University, Newcastle, United Kingdom; 3 Alcohol Health Network, London, United Kingdom; Cardiff University, UNITED KINGDOM

## Abstract

**Background:**

Most hazardous and harmful drinkers are of working age and do not seek help with their drinking. Occupational health services are uniquely placed to universally screen employees across the range of socioeconomic and ethnic groups. The aim was to explore the feasibility and acceptability of offering electronic screening and brief intervention for alcohol misuse in the context of a health check in six different workplace settings.

**Methods and Findings:**

Employees were recruited from six workplaces across England, including three local authorities, one university, one hospital and one petro-chemical company. A total of 1,254 (8%) employees completed the health check and received personalised feedback on their alcohol intake, alongside feedback on smoking, fruit and vegetable consumption and physical activity. Most participants were female (65%) and of ‘White British’ ethnicity (94%), with a mean age of 43 years (SD 11). Participants were mostly in Intermediate occupations (58%), followed by Higher managerial / professional (39%) and Routine and manual occupations (2%). A quarter of participants (25%) were drinking at hazardous levels (33% male, 21% female), which decreased with age. Sixty-four percent (n=797) of participants completed online follow-up at three months. Most participants were supportive of workplaces offering employees an online health check (95%), their preferred format was online (91%) and many were confident of the confidentiality of their responses (60%). Whilst the feedback reminded most participants of things they already knew (75%), some were reportedly motivated to change their behaviour (13%).

**Conclusions:**

Online health screening and personalised feedback appears feasible and acceptable, but challenges include low participation rates, potentially attracting ‘worried well’ employees rather than those at greatest health risk, and less acceptance of the approach among older employees and those from ethnic minority backgrounds and routine or manual occupations.

## Introduction

Alcohol misuse remains a global health concern, responsible for 5.9% (3.3 million) of deaths and 5.1% (139 million DALYs) of disease and injury in 2012, with the highest rates in the European Region [1]. Alcohol misuse is also an economic concern, with the cost of alcohol-related harm in Europe estimated at €155 billion (£122bn / US$195bn) in 2010, encompassing costs to the health service, workplace and criminal justice setting [2]. The World Health Organisation’s (WHO) global strategy for addressing alcohol-related harm advocates the provision of screening and brief intervention (SBI) for adults drinking at hazardous and harmful levels in primary care and other settings [3], where alcohol misuse is defined as drinking above recommended limits and incorporates hazardous (not experiencing harm), harmful (experiencing harm) and dependent drinking (experiencing symptoms of dependence) [4]. Brief interventions must be delivered at a population level to have an impact on public health, but this has not been achieved in the health care setting to date [5]. Two ways of broadening the reach of SBI is to deliver interventions over the Internet and in non-health care settings.

In Britain, 78% of the population aged 14 years and above use the Internet, with the biggest rise in the lowest income groups from 2011 to 2013 thereby narrowing the digital divide [6]. There is an emerging evidence-base for screening and brief intervention delivered by electronic devices (eSBI), particularly in student samples [7–13]. Computer-delivered interventions have been found to result in a reduction of 26 grams of alcohol per week (95% confidence interval (CI): −41 to −11) compared with minimally active comparator groups, such as assessment only and information-only websites [7]. Whilst this magnitude of effect is slightly less than that found with in-person SBI in primary care settings (mean difference: −38 grams of alcohol per week, 95% CI: −54 to −23] [14]), a sub-group analysis of non-student populations vs. student populations receiving computer-based interventions found significantly greater reductions in alcohol intake in the studies of non-students [7]. The bulk of the evidence for SBI and eSBI exists in primary care and higher educational settings respectively, yet other settings hold promise for maximising the reach of these interventions.

Most hazardous and harmful drinkers are of working age and do not seek help with their drinking. The workplace provides an opportunity for reaching population level penetration of SBI, with 73% of the working aged population (16 to 64) in employment in the UK in 2014 [15] and a higher proportion of working adults drinking in the past week (65%) than those unemployed (48%) in 2012 [16]. Occupational health services have the opportunity to universally screen individuals across a wide range of socioeconomic and ethnic groups. In 2009, a systematic review of workplace interventions for alcohol-problems identified four randomised controlled trials (RCTs), two from the US, one from Australia and one from Sweden [17]. This review found preliminary support for brief interventions and psychosocial skills training in the workplace; however, the studies reported several methodological challenges such as lack of exposure to the intervention and control groups receiving the intervention. Two of the studies included in this review used the Internet to deliver the intervention in US workplace settings [18, 19]. One study found online personalised normative feedback (Check Your Drinking) to be more effective at reducing alcohol intake than assessment-only [18]. The other study had difficulties with recruitment related to employees’ privacy concerns and provided only preliminary support for electronic screening and brief intervention in the workplace [19]. The perceived repercussions of divulging sensitive information may affect participation and self-reported veracity [20]. It is possible that employees will under-report their alcohol intake in the workplace setting, where there may be concerns over the confidentiality of their responses. There is a limited literature on how employees feel about answering questions on their health in the workplace. One way of tackling the stigma of accessing an intervention aimed specially at alcohol intake is to deliver the intervention alongside multiple risk behaviours as part of a healthy lifestyle intervention.

Screening and brief intervention for multiple risk behaviours, such as alcohol misuse, tobacco smoking, low levels of fruit and vegetable intake and physical activity have grown in popularity for a number of reasons: they can simultaneously address all of the leading contributors to preventable mortality and morbidity [21, 22], they use similar intervention approaches for each behaviour [23, 24] and they may minimise the stigma experienced by patients and practitioners when focusing on a single health behaviour [25, 26]. The National Health Service in England (NHS England) provides health checks to adults aged 40 to 74 every five years [27] and healthcare professionals are encouraged to ask about health behaviours in every consultation as part of the “Making every contact count” initiative [28], with a view to refocusing the NHS towards prevention and health promotion. Whilst the efficacy of interventions for individual health behaviours has been established, there is limited research on the impact of interventions that target multiple risk behaviours [29, 30]. Delivering health checks online have the added advantage of reach, convenience and consistently delivered content. A small number of studies have investigated the use of online interventions for multiple health behaviours including alcohol intake in different settings, such as students in primary care [31], adults from the general population [32] and workplace employees [33]. A recent trial (n = 5,055) of respondents to a population health survey in the Netherlands compared online tailored motivational feedback on multiple health behaviours (i.e. fruit and vegetable consumption, physical activity, smoking and alcohol intake) delivered sequentially or simultaneously. The trial found significant improvements in “overall lifestyle risk factor” (composite measure of compliance with Dutch guidelines) with the combined intervention groups compared with the control group who received criterion feedback [32], however findings for individual behaviours in sensitivity analyses were inconclusive. Another recent trial in the UK (n = 1,330) investigated the impact of an online health check that included personalised feedback on the same health behaviours as the Dutch trial above, but focused exclusively on its impact on alcohol intake among employees of a large private sector organisation of approximately 100,000 employees [33, 34]. Participants in this trial responded to an invitation to take part in an online health check, advertised on the company web-portal, and were drinking above recommended limits in England (i.e. scored five or more on Alcohol Use Disorders Test—Consumption (AUDIT-C) [35, 36]). The trial found no difference at three months in past week alcohol intake between participants receiving feedback on all health behaviours (intervention) and those receiving feedback on all behaviours except alcohol intake (control) [34]. That trial left many questions unanswered, such as the acceptability of the health check approach, privacy concerns around the Internet and workplace setting, usefulness of the feedback, and whether concerns differ by demographic characteristics and health behaviours. In view of the Medical Research Council guidance on optimising trial parameters before undertaking Phase 3 trials [37], we decided to undertake a single arm feasibility study to illuminate these issues and to explore whether there are differences between different types of organisations.

## Methods

### Aim

To explore the feasibility and acceptability of offering electronic screening and brief intervention for alcohol misuse in the context of a health check in six different workplace settings.

### Objectives

1. To undertake a feasibility study of online screening, personalised feedback and access to an extensive online alcohol intervention in six workplace settings in the North and South of England.
2. To determine whether a definitive multicentre trial is feasible by estimating study parameters and thereby informing a sample size calculation. Study parameters include rates of eligibility, recruitment and retention at three months.
3. To explore the acceptability to employees of completing an online health screen, receiving feedback on health behaviours and access to an online alcohol intervention, and completing follow-up measures, with particular reference to perceived risks to confidentiality.
4. To determine the extent of access to an extended online alcohol intervention, and its suitability for this population.

### Study design

A multi-site feasibility study of electronic screening and brief intervention for reducing alcohol intake in employees of six workplace settings in the North and South England. Ethical approval was granted by University College London Research Ethics Committee (4213/001). The study was registered with the UCL data protection officer. The protocol for this feasibility study is available as supporting information; see S1 Protocol.

### Setting

**Fig 1:**
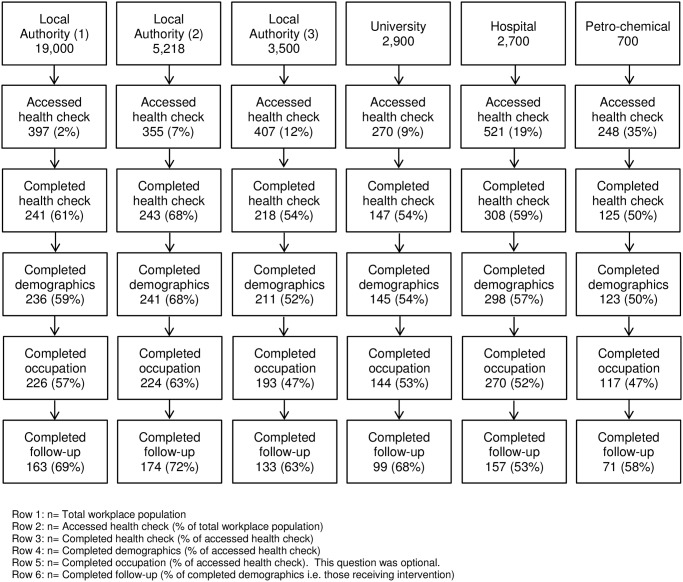
Strobe diagram of participant flow through the study.

Organisations were identified by one of the authors (DS) in the capacity of Director of the Alcohol Health Network, a social enterprise that offers support to workplaces with alcohol awareness campaigns. Six organisations were selected for their diversity, including five public sector and one private sector organisations, different types of public sector organisations (three local authorities, one hospital and one university), different sized organisations (ranging from 700 employees to 19,000) (see Fig. 1 Strobe diagram of participant flow through the study), different geographical regions (three local authorities and one private company in the North of England; one hospital and one university in the South of England) and both rural and urban areas.

### Participants

Eligible participants were employees at each of the six workplaces, providing informed consent. As employees of these companies, participants were adults with the ability to read English. Employees needed to gain access to the Internet to participate in this study.

### Recruitment

Occupational health leads in each organisation decided on the most suitable recruitment procedure for their organisation, e.g. email, Intranet, newsletter / magazine (electronic and hard copy), electronic noticeboard and posters. Employees were invited to complete an online health screen and to take part in a study led by researchers from UCL (University College London) and NCL (Newcastle University). If interested in learning more about the study, employees were invited to visit the study website. The study website provided information on what taking part involved, the potential advantages and disadvantages of participation and the implications of the findings. Participants were not informed of the specific alcohol focus of the study to minimise the stigma associated with participation and to minimise bias in the responses. It was made clear that taking part was voluntary and that their employer would not know whether they had taken part, and that all the information would be anonymised (i.e. not linked with their email address). If happy to take part, employees completed an online consent form and provided an email address to enable the research team to contact them again in three months’ time. On completion of the consent form, participants completed the health screen (as detailed below).

### Baseline data collection

#### Health screen

Participants were asked questions on a range of behaviours known to impact on their health and wellbeing, namely alcohol, smoking, diet and physical activity. The 3-item AUDIT-C questionnaire was used as an initial screen for alcohol misuse [35]. The AUDIT-C comprises the first 3 consumption questions of the WHO Alcohol Use Disorders Identification Test (AUDIT) [4]. Questions relate to the frequency of alcohol consumption, quantity of alcohol consumed on a typical drinking day, and frequency of drinking more than 6 (women) or 8 (men) drinks on one occasion. Participants scoring five or more on the AUDIT-C were presented with the remaining seven questions of the full 10-item AUDIT which focus on alcohol-related harm. Scores of eight or more on the full AUDIT indicate hazardous, harmful or dependent drinking, with high sensitivity (92%) and specificity (94%) [38]. Guidelines in England advise clinicians to use this level to detect alcohol misuse [36].

The health screen used the same approach as the health check offered in the Health on Web (HOW) trial [33, 34], asking employees about their smoking status; the average number of portions of fruit and vegetables they consumed per day, where the recommended number of portions in the UK is 5 or more; the average number of minutes spent undertaking light, moderate and vigorous activity a week, where more than 150 minutes of moderate or vigorous activity is recommended a week in the UK.

#### Demographics

Demographic data were collected at the end of the health screen before provision of feedback on health behaviours, and these included: gender, age, ethnicity and occupational classification (categories were supplied by the occupational health lead from each organisation). Provision of occupational classification was optional. These data were subsequently mapped to the three occupational categories detailed in the National Statistics Socio-economic Classification (NS-SEC) to allow comparison across workplaces: the three class classification includes: 1. Higher managerial, administrative and professional occupations, 2. Intermediate occupations, 3. Routine and manual occupations [39].

### Intervention

#### Healthy Choices Online tool

All participants providing demographic data received instantaneous personalised feedback from the health screen in-line with standard NHS recommendations for healthy living. Participants who scored less than five on the AUDIT-C or less than eight on the AUDIT, did not smoke, ate five or more portions of fruit and vegetables a day or undertook 150 minutes or more of moderate or vigorous physical activity a week were commended for leading a healthy lifestyle and reducing their risk of heart disease or cancer, for example. Participants who exceeded AUDIT thresholds, smoked, ate less than five portions of fruit and vegetables a day or did less than 150 minutes of moderate or vigorous physical activity a week received feedback on the government’s recommendations and the benefits of changing their behaviour. This brief advice was accompanied by hyperlinks to corresponding pages of the NHS Choices and NHS Livewell websites for further information, along with signposting to local services. Participants exceeding the AUDIT threshold received brief advice on the harms of excess drinking, along with a hyperlink to the Down Your Drink (DYD) website. The Down Your Drink website aimed to help adults from the general population to reduce their drinking. It was based on approaches known to be effective at motivating, eliciting and maintaining change, including motivational enhancement, cognitive behavioural therapy, and relapse prevention. The programme helps people reach high-quality decisions about whether to change their drinking; plan a specific change and provides the tools needed to cut down; and provides support with maintaining change and avoiding relapse [40]. As such, Down Your Drink is an extended online intervention and may or may not be needed or used by all employees. This was explored in the follow-up questions.

### Outcomes and outcome measures

All participants were contacted by email three months after baseline data collection to complete follow-up questionnaires online, via hyperlink, regardless of level of alcohol consumption at baseline. Outcome measures included the AUDIT and a questionnaire designed by the research team that explores the acceptability and potential risk of delivering electronic screening and brief intervention to employees in the workplace. The questionnaire also included questions on whether participants used the Down Your Drink website. The study protocol specified the following success criteria for progression to a definitive multicentre trial of eSBI in these workplace settings:

1. Eligibility: The definitive trial would only include adults scoring eight or more on the AUDIT. According to population survey data, the proportion of adults drinking above recommended limits in England is 25%. We would therefore only progress to a definitive trial if at least 25% of participants meet this criteria.
2. Recruitment: rates equal or above 5% of the total adult workforce eligible to take part in this study in at least one organisation. This was based on the recruitment rate of 3% in a randomised controlled trial that evaluated a similar intervention in a large, UK-based workplace setting [34].
3. Retention: 50% at 3-month follow-up in at least one organisation. This is based on an online trial of DYD [41], where email prompts at 3-months obtained a follow-up rate of 48%. The data in this study are collected online and prompted by email alone, which yields lower response rates than a combined approach with postal reminders and telephone calls.

If all of the above criteria are met the authors will proceed to a definitive trial. Decisions over whether to modify the protocol will be informed by responses to the questions on acceptability, potential risk and use of DYD.

### Analyses

Baseline data on demographics and health behaviours are presented as numbers with percentages and median with interquartile range or mean (SD). Data from the follow-up questionnaires on acceptability of the health check are presented as numbers and percentages answering each of the multiple choice questions. Odds ratios and 95% confidence intervals were calculated for the following variables: gender (male, female), age (18–29, 30–39, 40–49, 50–59, 60+), ethnicity (‘White’ and ‘non-White’ ethnicity) and AUDIT score (≥8 or less than 8).

## Results

### Recruitment

A total of 2,198 employees accessed the online health check across the six workplaces in July and August 2013 for a period of three months. The response rate varied from 2% to 12% in the three local authorities, 9% in the university, 19% in the hospital and 35% in the petro-chemical company (see Fig. 1 Strobe diagram of participant flow through the study). A total of 1,254 employees received the intervention (having completed the health check and demographic information), comprising 57% of employees who initially accessed the health check across all worksites: 52% to 68% in the three local authorities, 54% in the university, 57% in the hospital and 50% in the petro-chemical company (see Fig. 1 Strobe diagram of participant flow through the study).

### Retention

A total of 797 (64%) participants completed the AUDIT at three month follow-up after up to three email prompts, which ranged from 63% to 72% in the three local authorities, 68% in the university, 53% in the hospital and 58% in the petro-chemical company (see Fig. 1 Strobe diagram of participant flow through the study). The following participants were more likely to complete follow-up questionnaires than not complete them: participants of ‘White’ ethnicity (OR 1.73; 95% CI: 1.06, 2.83), participants aged 40–49 (OR 1.88; 95% CI: 1.31, 2.72) and aged 50–59 (OR 2.06; 95% CI: 1.42, 2.99) compared with those aged 18–29. Whereas, the following participants were less likely to complete follow-up questionnaires than complete them: participants working in the hospital (OR 0.52; 95% CI: 0.39, 0.68) compared with those who worked for a local authority, participants scoring eight or more on the AUDIT (OR 0.68; 95% CI: 0.52, 0.89) and participants who smoke (OR 0.57; 95% CI: 0.39, 0.83).

### Baseline characteristics

#### Demographics

Participants across all workplaces were mostly female (65%; range 56% to 79%), with the exception of the petro-chemical company (18%) (see Table 1 for baseline characteristics). The mean age across all workplaces was 43 years (SD 11) and the majority of participants were of ‘White British’ ethnicity (94%). Of the ‘non-White’ participants, most were from the London based university (21%) and hospital (69%), as opposed to the other worksites based in the North of England (10% from x3 local authorities and petro-chemical company). Participants were classified as having largely Intermediate occupations (58%), followed by Higher managerial / professional (39%) and Routine and manual occupations (2%). Most participants were based in the three Local authorities (55%), followed by the hospital (24%), university (12%) and the petro-chemical company (10%). More participants were based in the North of England (65%).

**Table 1:** Baseline characteristics across all six workplaces.

Variable	Baseline	
	n/N or median	% or (IQR)
Age mean (SD)	43	(11)
Age 18–29	189/1254	15
Age 30–39	290/1254	23
Age 40–49	357/1254	28
Age 50–59	337/1254	27
Age 60+	81/1254	6
Male	439/1254	35
White	1179/1254	94
Higher managerial/ professional	442/1123	39
Intermediate occupations	653/1123	58
Routine and manual occupations	28/1123	2
Organisation		
Local Authority (1)	236/1254	19
Hospital	298/1254	24
University	145/1254	12
Local Authority (2)	241/1254	19
Petro-chemical	123/1254	10
Local Authority (3)	211/1254	17
Workplace in the north	811/1254	65
Workplace type		
Local Authority	688/1254	55
Higher education institute	145/1254	12
Hospital	298/1254	24
Private company	123/1254	10
AUDIT 8+	312/1254	25
AUDIT	4	(3, 7)
AUDIT lower risk	942/1254	75
AUDIT increasing risk	268/1254	21
AUDIT higher risk	28/1254	2
AUDIT dependent	16/1254	1
Smoker	121/1254	10
Number smoked per day (in smokers only)	10	(5, 15)
<5 portions of fruit or vegetables/ day	876/1254	70
Portions of fruit or vegetables/ day	3	(2, 5)
<150 minutes moderate or vigorous physical activity per week	182/1254	15
Minutes of physical activity per week	350	(210, 560)

#### Health behaviours

The median AUDIT score across all respondents in all workplaces was 4 (IQR 3, 7) (male 5 IQR 3, 7; female 4 IQR 2, 6). The proportion of participants scoring eight or more on the AUDIT was 25% across all workplaces (33% male, 21% female). The proportion of participants scoring eight or more on the AUDIT decreased with age: 41% 18–29, 30% 30–39, 21% 40–49, 19% 50–59, 14% 60+. The proportion of participants scoring eight or more on the AUDIT was higher in ‘White British’ participants compared with ‘non-White’ participants (26% vs. 15%), and slightly higher in Routine and manual occupations (29%) compared with Higher managerial / professional (25%) and Intermediate occupations (25%). The proportion of participants scoring eight or more on the AUDIT ranged from 23% to 28% in the three local authorities, 25% in the university, 18% in the hospital and 34% in the petro-chemical company. The proportion of AUDIT positive participants was higher among employees in the North of England (27%) compared with the South (21%).

Prevalence of smoking among employees across all workplaces was 10%, median level of physical activity was 350 (IQR 201, 560) minutes a week (with 85% exceeding recommended threshold) and median fruit and vegetable consumption was 3 portions a day (with 30% exceeding threshold).

### Acceptability of the online health check

The vast majority of participants who completed follow-up questionnaires at three months were supportive of workplaces offering employees an online health check (95%), which was largely consistent across demographic groups and different health behaviours (see Table 2). Similar levels of acceptance were reported for the inclusion of questions on tobacco, alcohol and food consumption and level of physical activity, with employees slightly more reluctant to report illicit drug use. Participants of ‘White’ ethnicity were less likely to mind the inclusion of alcohol in the health check than ‘non-White’ participants (91% vs. 77%; OR 2.59 95% CI 1.09, 6.18). Employees’ preferred format for the delivery of the health check was online / email (91%), although this was slightly lower in Routine and manual occupations (83%), compared with Higher managerial / professional (92%) and Intermediate occupations (91%).

**Table 2:** Acceptability of using online health check at three month follow-up.

Variable	n/N	%
Workplaces should offer staff/ employees health checks if they want one	733/775	95
Would respondents mind if health checks included:		
Tobacco consumption	707/797	89
Alcohol consumption	720/797	90
Food consumption	732/797	92
Physical activity	733/797	92
Recreational or illicit drugs	644/797	81
Preferred delivery method of health check		
Online/ email	704/775	91
Paper posted to your home address	40/775	5
Paper at your work address	31/775	4
Concerns about an online health check		
Employer may see the results—not concerned	552/775	71
Employer may see the results—neutral	107/775	14
Employer may see the results—concerned	116/775	15
Colleagues may see the results—not concerned	543/775	70
Colleagues may see the results—neutral	99/775	13
Colleagues may see the results—concerned	133/775	17
Personal information on the internet—not concerned	336/775	43
Personal information on the internet—neutral	233/775	30
Personal information on the internet—concerned	206/775	27
Completing it in work time—not concerned	572/775	74
Completing it in work time—neutral	121/775	16
Completing it in work time—concerned	82/775	11
Feedback will not be accurate—not concerned	455/775	59
Feedback will not be accurate—neutral	190/775	25
Feedback will not be accurate—concerned	130/775	17
Read the feedback that was provided following completion of the health check	709/762	93
The feedback was a complete waste of time—disagree	422/762	55
The feedback was a complete waste of time—neutral	267/762	35
The feedback was a complete waste of time—agree	73/762	10
The feedback reminded me of things I knew already—disagree	58/762	8
The feedback reminded me of things I knew already—neutral	132/762	17
The feedback reminded me of things I knew already—agree	572/762	75
The feedback surprised me—disagree	517/762	68
The feedback surprised me—neutral	220/762	29
The feedback surprised me—agree	25/762	3
The feedback motivated me to change—disagree	284/762	37
The feedback motivated me to change—neutral	379/762	50
The feedback motivated me to change—agree	99/762	13
In general, providing feedback is a good idea, but it wasn’t helpful to me personally—disagree	179/762	23
In general, providing feedback is a good idea, but it wasn’t helpful to me personally—neutral	268/762	35
In general, providing feedback is a good idea, but it wasn’t helpful to me personally—agree	315/762	41
Confidence of confidentiality		
Confident	453/755	60
Not sure	288/755	38
Convinced my employer will find out	14/755	2
Internet proficiency		
Experienced	701/755	93
Occasional user	54/755	7
First use		
Method of accessing assessment		
Work computer	658/755	87
Home computer	59/755	8
Mobile phone	20/755	3
Tablet	10/755	1
Other	8/755	1
Information would be willing to give to a researcher		
Telephone number	176/797	22
Home address (for letters)	224/797	28
Accessed the DYD website	35/749	5
If not why?		
Don’t want to change my drinking	256/797	32
Don’t want to use a website to think about my drinking	37/797	5
Didn’t look interesting	31/797	4
Don’t have time	133/797	17
Other	268/797	34
If yes, it provided the information I was looking for	25/40	63
Types of alcohol-related resources accessed in the past three months		
Websites	22/797	3
Self-help leaflets or books	12/797	2
Telephone helpline	0/797	0
GP	5/797	1
Counselling services	6/797	1
Other	80/797	10

### Concerns about the online health check

Overall, most employees reported not being concerned about their employers (71%) or colleagues (70%) seeing the results of the health check. Those scoring eight plus on the AUDIT were more likely to report being concerned about their employers seeing their health check than those scoring less than eight (22% vs. 13%; OR 2.12 95% CI: 1.36, 3.30). Those scoring eight plus on the AUDIT also reported greater concern about their colleagues seeing their health check compared with those scoring less than eight (24% vs. 15%; OR 1.75 95% CI: 1.14, 2.68). Most participants were either not concerned (43%) or felt neutral (30%) about providing personal information on the Internet. Reported concern was greatest among the participants aged 60 years and above (46%; OR 2.16 95% CI 1.57, 2.97) compared with other age groups, among ‘non White’ ethnicities (53% vs. 25%; OR 0.21 95% CI 0.08, 0.53) and in Routine and manual occupations than Higher managerial / professional and Intermediate occupations (50% vs. 27%, 25%). Most employees reported not being concerned about completing the health check during work time (74%). Participants of ‘non-White’ ethnicity reported more concern about completing the health check during work time than those of ‘White’ ethnicity (22% vs. 10%; OR 0.31 95% CI 0.12, 0.81). Fifty nine percent of participants reported not being concerned about the feedback not being accurate, whereas 17% of participants were concerned. Concern was greatest among participants aged 60 years and above (27%; OR 1.56 95% CI: 1.09, 2.22). Concern about the feedback not being accurate was also higher in Routine and manual occupations.

### Impact of health check

The vast majority of participants reported reading the feedback provided by the health check (93%). Over half (55%) of participants did not feel that the feedback was a complete waste of time, 35% were neutral, whereas 10% of participants did feel that the feedback was a waste of time. Twenty four percent of those aged 60 years and above thought the feedback was a waste of time (OR 2.19 95% CI: 1.39, 3.46 compared with other age groups). For most participants, the feedback reminded them of things they already knew (75%), which was higher in Higher managerial / professional and Intermediate occupations than Routine and manual occupations. Eight percent of participants reported that the feedback was new to them. Most participants felt that the feedback did not surprise them (68%), whereas 3% felt that the feedback had surprised them. Participants scoring eight or more on the AUDIT reported being more surprised at the feedback than those scoring less than eight (6% vs. 3%; OR 2.58 95% CI: 1.12, 5.92). When asked if the feedback motivated them to change, half provided a neutral response (50%), followed by 37% who disagreed with the statement and 13% who agreed.

### Internet setting

Sixty percent of participants were confident that their responses to the health check were confidential, with 2% convinced their employer would find out. Participants scoring eight or more on the AUDIT were less confident of confidentiality than those scoring less than eight (47% vs. 35%; OR 1.72 95% CI 1.21, 2.45). Most participants (93%) reported to be experienced users of the Internet. Occasional users of the Internet were more likely to be aged 50 years and above (14% 50–59, 13% 60+). Most employees accessed the health check via their work computer (87%), which varied by ethnicity (‘non-White’ 61% vs. 88% ‘White’) and region (South of England 78% vs. North of England 91%).

### Accessed down your drink

A small number of participants (5%) reported accessing the Down Your Drink website, most of whom were 60 years and above, all were of ‘White’ ethnicity and most were university employees. Ten percent of participants scoring eight or more on the AUDIT at baseline accessed DYD compared with 3% of participants scoring less than eight. Of those accessing DYD, 63% (50% male, 71% female) said it provided the help they were looking for. The main reason for not accessing DYD was because participants did not want to change their drinking (39% male vs. 29% female; OR 1.5 95% CI: 1.08, 2.97). Younger participants were more likely to state lack of time as a barrier than older participants (21% 18–29 year olds, 21% 30–39 year olds).

### Use of other resources

Small numbers of participants reported accessing other alcohol-related resources over the past three months including: websites (3%), self-help leaflets / books (2%), GP (1%), or counselling services (1%).

## Discussion

This study found that the delivery of an online health check appears feasible, as recruitment, eligibility and retention rates met the pre-determined success criteria for progression to a definitive trial. The study found that most participants were supportive of workplaces offering employees an online health check, their preferred format was online and many were confident of the confidentiality of their responses. Whilst the feedback reminded most participants of things they already knew, some were reportedly motivated to change their behaviour. A small number of participants drinking at hazardous levels accessed further support with their drinking via the Down Your Drink website. Important considerations for further research on the delivery of an online health check in workplace settings include, small proportions of total workplace employees participating and less acceptance of the intervention among older participants, those of ‘non-White’ ethnicity and among Routine and Manual workers. Online health checks for delivery in the workplace should be evaluated as part of a multi-faceted approach, which meets the needs and preferences of a diverse workforce.

### Recruitment

Recruitment rates varied considerably from 1% to 18% of the total workforce, with an average rate of 8% over all six organisations. These rates are low compared with a feasibility study of in-person brief alcohol intervention in a Local Authority in Scotland (approximately 7,500 employees), which recruited 627 (41%) of a pre-identified sample of employees to complete a paper-based survey on health and lifestyle behaviours (including the AUDIT) [42]. This study did however, incentivise employees with a prize draw for £50 department store voucher. In one of the most recent trials of online brief alcohol intervention, delivered across seven universities in New Zealand, 5,135 (34%) students completed screening following email advertisements [43]. Some of the organisations in our study used email to directly advertise the online health check to employees (hospital and Local Authority (2)), but this did not appear to be associated with the highest rates of recruitment. The highest recruitment rate was found in the private sector organisation, which might be explained by the corporate culture and more stringent alcohol policy in this type of workplace setting, whereby employees have a dual responsibility for undergoing health checks. The organisations were provided with posters and flyers/leaflets to advertise the online health check, which were adopted by all except one organisation which preferred to design their own. The exact location and extent of distribution of the recruitment material was determined by the organisation. It is important to note that of those employees accessing the intervention, between 50% and 68% of these respondents actually completed the health check and demographic information and received the personalised feedback. Once accessing the health check, employees may have been deterred by the request for a personal email address, by taking part in a research study (although this was mentioned in the advertisement) or by completion of a consent form. Of those employees who completed the health check and demographic information, five of the six organisations met the 5% threshold defined in our protocol for progression to a definitive trial. Before pursuing a definitive trial, qualitative research should explore the barriers to completing an online health check in different types of workplace settings, delivered as part of a research study, in addition to exploring the impact of different recruitment strategies.

### Participant characteristics

Participants in this study were self-selected; they responded to an advertisement to take part in an online health check as part of a research study, rather than being referred to it. The study largely attracted healthy employees compared with the general adult population in England, with low smoking prevalence (10% vs. 20% in 2010 [44]), high levels of physical activity (85% exceeding recommendations vs 61% in 2012 [45]) and comparable numbers consuming five or more portions of fruit and vegetables a day (30% vs. 31% in 2012 [45]). The health check used the AUDIT measure of alcohol consumption and alcohol-related harm to screen employees for alcohol misuse, with 25% of employees exceeding the threshold and thus meeting the pre-specified eligibility criteria for progression to a definitive trial. Population data on the AUDIT was last collected in England in 2007 and found a similar proportion of people exceeding the threshold (24% [46]). The proportion of participants drinking above the AUDIT threshold was similar to the Watson study (24%), despite restricting inclusion to hazardous drinkers with AUDIT scores of 8–15 for men and 6–15 for women [42]. The online health check offered in these six organisations has to some extent attracted the ‘worried well’. The key challenge facing further research in this field is how to engage those most likely to benefit, i.e. those exhibiting unhealthy behaviours. It is possible that the context of the workplace discourages those most in need to take part, with possible perceived repercussions on careers, particularly related to disclosing alcohol consumption. Or perhaps the offer of a health check itself is more likely to attract healthier people. Future research would benefit from comparing the online health check with an alcohol-only intervention to see which approach attracts a greater proportion of people drinking at hazardous and harmful levels. A randomised trial comparing in-person alcohol-only vs. multiple health behaviour intervention is currently underway in a UK general hospital setting [47].

### Retention

The number of participants completing follow-up questionnaires at three months ranged from 53% to 72%, thus meeting the success criteria of 50% in at least one organisation. This study followed up participants via email alone due to time and resource constraints. The HOW trial achieved an 80% response rate, where email prompts were supplemented with postal requests for follow-up and telephone prompts. Few participants in this study reported being willing to provide researchers with a telephone number (22%) or postal address (28%) for the purpose of follow-up. The question for future trials in this setting is to what degree is a pragmatic approach is required. Restricting inclusion to those participants who provide an email address, postal address and telephone number is less pragmatic in that it does not mirror the way in which employees would access the online health check if made available outside a research study, however, more intensive follow-up would improve the internal validity of the trial by achieving higher rates of follow-up.

### Acceptability

The vast majority of participants (95%) were supportive of workplaces offering employees an online health check, however it is important to bear in mind participants were those who wanted to complete one. An important, but not surprising finding is that more participants exceeding the AUDIT threshold were more concerned about their employers and other employees seeing their feedback than those scoring less than eight, which may have deterred some employees from taking part in the study. As mentioned above, a key challenge to delivering an online health check intervention in the workplace setting is to attract those most likely to benefit; this could be achieved by making completion compulsory, however there may then be implications on the veracity of the reported data. An online health check may not be best suited to older employees (aged 60 years and above), those of ‘non-White’ ethnicity and those in Routine and manual occupations, who were most concerned about providing personal information on the Internet and had less confidence in the accuracy of the feedback. Alcohol consumption in the over 60s is a growing concern in England, with older people drinking more frequently than younger people [48] and accounting for 50% more alcohol-related hospital admissions for mental and behavioural disorders [49]. Nevertheless, older employees were more likely to access the Down Your Drink website for more information and support with changing their drinking. Cultural differences may exist around the acceptability of accessing a non-work related resource in work time. These are important issues to explore in further research, as are interventions best suited to the needs of Routine and Manual employees.

### Strengths and limitations

One of the advantages of conducting research into brief alcohol interventions in the workplace setting is the demand from both public and private sector organisations for interventions aimed at tackling alcohol misuse. The occupational health leads involved in this study were enthusiastic about providing a free online intervention for their employees; this is possibly a result of Prof Dame Carol Black’s, Working for a Healthier Tomorrow report, which advocates the benefits of healthier workforces for the economy and business [50]. To further engage occupational health leads, they were asked to select the measure of occupational classification for their organisation and to determine how and where recruitment materials should be distributed to reach all employees. Whilst this is seen as a strength of this study, occupational categories were later mapped onto the three class National Statistics Socio-economic Classification to enable comparison across workplaces on the acceptability of the health check, and this process may have introduced some inaccuracies in group assignment. This study does not tell us whether the demographics and health behaviours of participants in each organisation represent those of each company as a whole. The online health check attracted a small number of participants (2%) in Routine and manual occupations, which may not reflect the proportion of these employees in these organisations. This study found participants in Routine and manual occupations slightly less supportive of the Internet for delivering the health check and were more concerned about providing personal information and the accuracy of the feedback, however, this is a tentative interpretation due to the small numbers of participants in this occupational category and further exploration of the barriers and facilitators to accessing health behaviour interventions among this occupational group in the workplace should be explored.

## Conclusions

Promotion of an online health check in workplace settings appeared to attract relatively healthy individuals, with lower smoking prevalence, higher levels of physical activity than the general population, and with levels of alcohol intake and fruit and vegetable consumption comparable to the general population. This may help explain the finding that an online health check approach was largely seen as acceptable among employees in this study, but further research should focus on a more suitable approach for adults over 60, ‘non-White’ ethnicities and Routine and manual workers. Future research in the workplace needs to focus efforts on recruiting much larger proportions of the total population.

## Supporting Information

S1 Protocol Feasibility study of electronic screening and brief intervention for alcohol misuse in workplace settings. (DOCX)
