# Supplementary material for: Online Health Check for Reducing Alcohol Intake among Employees: A Feasibility Study in Six Workplaces across England
Source: PLoS One. 2015 Mar 23;10(3):e0121174. doi: 10.1371/journal.pone.0121174 (PMC4370494; doi:10.1371/journal.pone.0121174)
Supplement: S1 Protocol — (DOCX) [file pone.0121174.s001.docx]

**Feasibility study of electronic screening and brief intervention for alcohol misuse in workplace settings**

**Protocol**

**Background**

*Alcohol-related harm and cost to the workplace*

Alcohol misuse (drinking above safe limits) is among the leading causes of preventable mortality and morbidity worldwide, with the highest death rates found in the European region ([1](#_ENREF_1)). In England in 2010, almost a quarter of adults (22%) aged 16 and above were drinking more than recommended weekly limits (26% men and 17% women) ([2](#_ENREF_2)), although a recent comparison of this survey data with sales data suggests around three quarters of the population are drinking above safe limits ([3](#_ENREF_3)). Alcohol-related harm is not limited to the individual drinker, it affects other people, society and the economy, making it the most harmful drug in the UK ([4](#_ENREF_4)). An estimated 11 to 17 million working days are lost annually in England due to alcohol-related sickness, with an additional 15 to 20 million days lost due to reduced employment. Lost productivity (caused by alcohol-related absenteeism, unemployment and premature death) costs the country up to £6.4 billion ([5](#_ENREF_5)), with total costs of alcohol-related harm to the health service and wider economy of £20 billion a year ([6](#_ENREF_6), [7](#_ENREF_7)). A recent study in the US has estimated a 4:1 benefit to cost ratio for organisations that implement screening, brief intervention and referral to treatment to address alcohol issues at work over 4 years ([8](#_ENREF_8)).

*Screening and brief intervention in a workplace setting*

Brief interventions are effective at reducing alcohol intake and are advocated by NICE as the first step in a tiered service for alcohol misusers ([9](#_ENREF_9)). However, the evidence to support their use is largely based on brief interventions delivered by health care professionals in primary care settings ([10-15](#_ENREF_10)). For brief interventions to comprise the population level approach needed to tackle alcohol misuse, the entire population needs to be screened ([16](#_ENREF_16)).

There are numerous barriers to implementing screening and brief intervention (SBI) in primary care ([17-21](#_ENREF_17)) and so it is important to consider the effectiveness of SBI in other settings, as recommended by NICE ([9](#_ENREF_9)). Most hazardous and harmful drinkers are of working age and do not seek help with their drinking, making the workplace an ideal setting for SBI, with access to individuals across a range of socioeconomic groups and ethnicities. SBI for alcohol misuse should be an integral part of health promotion activity within occupational health departments. SBI was found to be acceptable in a workplace setting (Scottish Local Authority), where 92% of respondents to a general lifestyle survey were happy to be asked about their drinking ([22](#_ENREF_22)). In 2009, a systematic review of workplace interventions for alcohol-problems identified four randomised controlled trials (RCTs), two from the US, one from Australia and one from Sweden. Although there was some indication that brief interventions and psychosocial skills training are effective in this setting, studies were fraught with methodological limitations including lack of exposure to the intervention, contamination of the intervention and controls obtaining access to the intervention ([23](#_ENREF_23)). The workplace setting presents barriers to delivering SBI, which will differ across organisations, thus introducing methodological challenges to conducting a trial, and suggesting the need for a feasibility study as a precursor to designing a definitive randomised controlled trial in this setting.

*Role of occupational health in tackling alcohol misuse*

The World Health Organisation recommends the provision of personal health resources, as one of the four ways of achieving a healthy workplace, which may include healthy food choices, campaigns to encourage physical activity, no smoking policies and health assessments ([24](#_ENREF_24)). There is some evidence on the effectiveness of health resources delivered in the workplace but this largely concentrates on physical activity (for which there is NICE guidance specifically for this setting ([25](#_ENREF_25))), diet and smoking ([24](#_ENREF_24)). The challenges facing SBI for alcohol misuse in the workplace setting may explain the lack of research in this field. Alcohol consumption is a sensitive topic. The perceived repercussions of divulging sensitive information in-person may affect self-reported veracity ([26](#_ENREF_26)). It is possible that employees will under report their alcohol intake in the workplace setting, where there may be concerns over the confidentiality of their responses. There is a limited literature on how employees feel about answering questions on their health in the workplace. There may be concerns that the information they provide may impact on their employment thus deterring employees from participating in such activities, or leading them to report less than they are actually drinking. Using the Internet to confidentially deliver screening and brief intervention may tackle some of the concerns employees may have with SBI in this setting.

*Online interventions for reducing alcohol intake*

The internet is increasingly used to deliver SBI with its advantages of scalability, instantaneous access, flexibility of use, and anonymity, which is important for a stigmatised behaviour like alcohol misuse. There is a growing evidence-base for these interventions, particularly in student samples, which has been demonstrated in the large number of systematic reviews in this field ([27-33](#_ENREF_27)). A recent systematic review of computer-based interventions for reducing alcohol intake found them to be as effective as in-person interventions, such as CBT and motivational interviewing, in a small number of studies ([27](#_ENREF_27)). Two of the 24 studies included in this review (but not in the meta-analyses) were based in a US workplace setting ([34](#_ENREF_34), [35](#_ENREF_35)). One study found personalised normative feedback (Check Your Drinking) to be more effective at reducing alcohol intake than assessment-only ([34](#_ENREF_34)). The other study had difficulties with recruitment related to employees’ privacy concerns and provided only preliminary support for electronic screening and brief intervention (eSBI) in the workplace ([35](#_ENREF_35)).

The Health on the Web (HOW) study, an online trial of eSBI for alcohol misuse, is currently underway in a large private organisation with almost 100,000 employees ([36](#_ENREF_36)). Employees were invited to take part in an online health check (including questions on alcohol consumption, smoking, diet and exercise) via the company web portal. A total of 3,375 participants completed the health screen in a three week period, 39% of which were drinking above recommended safe limits (scored five or more on the AUDIT-C) and as such were randomised to receive feedback on all health behaviours including alcohol consumption or feedback on all health behaviours except alcohol. All employees had access to the company web portal, therefore ensuring equity of access. A follow-up rate of 80% was achieved with email, postal and telephone reminders. The organisation had an active occupational health team that organised quarterly health campaigns for their staff, which were aimed at prevention and early identification rather than treatment. Campaigns included online information, facilitated monitoring activity, risk assessment, prizes for winners of competitions, road shows, health fairs, articles in the company newsletter and email reminders to take part. The organisation had worked with other academic institutions, which meant its employees were used to taking part in research and answering questions on their health. Other organisations, particularly in the public sector where less funding is available for health promotion activity, may not be as successful at recruiting and retaining participants to a study of this nature. Study participation may also be less acceptable in occupational settings where employees are not used to being asked questions about their health.

The proposed study draws on two innovations in brief intervention research: 1) the occupational health setting and 2) electronic interventions, both of which provide the opportunity to increase the scalability of SBI and a step towards a public health approach to tackling alcohol misuse. The few studies that have been conducted in this field to-date point to the challenges of providing SBI and eSBI in the workplace setting. This feasibility study will explore the barriers to delivering eSBI across six workplace settings. It will also determine the potential barriers to conducting a trial in this setting, for example whether the study process and the intervention are acceptable to employees, whether the intervention is suited to the level of need or if alternative resources are more appropriate. This feasibility study of eSBI in the workplace forms the preparatory work needed for a multi-site RCT and has been designed to inform an application to NIHR Public Health Research for a definitive trial to address the question ‘Is eSBI effective in workplace settings?’

**Aim**

To explore the feasibility and acceptability of offering electronic screening and brief intervention (eSBI) for alcohol misuse in workplace settings. This study will inform the design of a multi-centre randomised controlled trial of the effectiveness of eSBI in this setting.

**Objectives**

1. To undertake a feasibility study of online screening, personalised feedback and access to an extensive online alcohol intervention in six workplace settings in the South and North East of England.

2. To determine whether a definitive multicentre trial is feasible by estimating study parameters and thereby informing a sample size calculation. Study parameters include rates of eligibility, recruitment and retention at three months.

3. To explore the acceptability and potential perceived risk to employees of completing an online health screen, receiving feedback on health behaviours and access to an online alcohol intervention, and completing follow-up measures.

4. To determine the level of engagement with an extensive online alcohol intervention, and its suitability for this population.

**Study design**

A multi-site feasibility study of electronic screening and brief intervention for alcohol misuse for employees in six workplace settings in the South and North East of England.

**Ethical approval and research governance**

Ethical approval will be sought from UCL research ethics committee for all workplaces. Letters of approval are needed from each of the employers to support the ethics application. UCL will also be the sponsor of this research.

**Setting**

Don Shenker, director of the Alcohol Health Network, has approached several organisations across England with the offer of electronic screening and brief intervention for their employees as part of a feasibility study led by UCL and NCL. There has been considerable interest from both public and private sector organisations, which demonstrates, to some extent, the acceptability of this approach with employers and the need for this type of intervention in the workplace. This feasibility study has restricted inclusion to six organisations, which will allow for an exploration of differences between organisations in the number of eligible participants, mechanisms for recruiting participants and differences in acceptability among employees. Exploration of these factors will help inform the design of a definitive multicentre trial. Inclusion of more than six organisations was considered unethical due to the nature of a feasibility study, where the effectiveness of the intervention is yet to be established in this setting and is not the purpose of the study. The following organisations were selected for their diversity, as they include both public and private sector organisations, different types of public sector organisations (council, hospital, university), different sized organisations (ranging from 700 employees to 19,000) and different geographical regions (South and North East of England, rural and urban areas):

- Hospital (2,700 employees)
- University (2,900 employees)
- Local authority 1 (19,000 employees)
- Local authority 2 (5,218 employees)
- Local authority 3 (3,500 employees)
- Petrochemical manufacturer (700 employees)

Details of the health promotion activities within each organisation will be provided by the occupational health leads. Occupational health leads will update the study team on previous, current and future health promotion campaigns at steering group meetings.

**Participants**

Eligible participants are employees at each of the six workplaces, providing informed consent. As employees of these companies, participants will be adults with the ability to read English. Employees will need to gain access to the Internet to participate in this study.

**Recruitment**

Occupational health leads will be consulted on the most suitable recruitment procedure for their organisation. Recruitment methods include: email, article on company web-portal or newsletter, or via posters. Occupational health leads will be asked to provide the reasons why they chose their preferred methods. Employees will be invited to complete an online health screen and to take part in a study led by researchers from UCL and NCL. If interested in learning more about the study, employees are invited to visit the study website, via hyperlink within emails, on web-portals or electronic newsletters. The study website will present the employee with participant information pages, which will explain the aim of the study, what will be involved if taking part, the potential advantages and disadvantages of participation and the implications of the findings. It will be made clear that taking part is voluntary, that their employer will not know whether they have taken part, and that all the information they provide will be anonymised (i.e. not linked with their name). If happy to take part, employees will be asked to complete an online consent form. On completion of the consent form, participants will be asked to complete the health screen (as detailed below).

**Baseline data collection**

*Health screen*

Participants will be asked to answer questions of a range of behaviours known to impact on their health and wellbeing, namely alcohol, smoking, diet and physical activity. The 3-item AUDIT-C questionnaire will be used as an initial screen for alcohol misuse ([37](#_ENREF_37)), with a score of five or more indicating consumption above recommended limits ([36](#_ENREF_36)). The AUDIT-C comprises the first 3 consumption questions from the WHO Alcohol Use Disorders Identification Test (AUDIT) ([38](#_ENREF_38)). Questions relate to the frequency of alcohol consumption, quantity of alcohol consumed on a typical drinking day, and frequency of drinking more than 6 (women) or 8 (men) drinks on one occasion. For participants scoring 5 or more on the AUDIT-C, the remaining seven questions of the AUDIT will then be presented which focus on alcohol-related harm. Questions on smoking status, consumption of fruit and vegetables and level of physical activity will also be included in the health screen, using the same approach as the HOW study ([36](#_ENREF_36)).

*Demographics*

Participants will be asked to provide demographic information, consisting of gender, year of birth, ethnicity, occupational classification (categories to be supplied by the occupational health lead from each organisation). Participants will also be asked to provide an email address that will allow them to be contacted again in three months’ time. Demographic data will be collected at the end of the health screen, before provision of feedback on health behaviours.

**Intervention**

All participants will receive instantaneous feedback from the health screen in-line with standard NHS recommendations for healthy living. Participants who drink within recommended limits, do not smoke, eat five or more portions of fruit and vegetables a day or undertake 150 minutes or more of moderate or vigorous physical activity a week will be congratulated for leading a healthy lifestyle and reducing their risk of heart disease or cancer, for example. Participants who drink above recommended limits, smoke, eat less than 5 portions of fruit and vegetables a day or do less than 150 minutes of moderate or vigorous physical activity a week will receive feedback on the governments recommendations and the benefits of changing their behaviour (to reduce their risk of harm). This brief advice will be accompanied by hyperlinks to corresponding pages of the NHS Choices and NHS Livewell websites for further information. Participants drinking above recommended limits will receive brief advice on the harms of excess drinking, along with a hyperlink to the Down Your Drink website. The Down Your Drink website is for people wanting to determine how much they are drinking, whether it is a problem for them and for support with making changes. Down Your Drink is an extensive online brief intervention and may or may not be used or needed by all employees. This will be explored in the follow-up questions.

*The Down Your Drink website*

Down Your Drink is aimed to help adults from the general population to reduce their drinking. It was developed and evaluated by a team based at UCL, with funding from the AERC and NPRI. DYD is based on approaches known to be effective at motivating, eliciting and maintaining change, including motivational enhancement, cognitive behavioural therapy, and relapse prevention. The programme helps people reach high-quality decisions about whether to change their drinking; helps plan a specific change and provides the tools needed to cut down; provides support with maintaining change and avoiding relapse. DYD also contains a number of ‘e-tools’, central to which is the ‘Drinking Episode Diary’. The diary allows users to record their alcohol consumption and their thoughts and feelings surrounding each drinking episode. The Drinking Episode Diary also calculates the cost and calories related to consumption each day. Other interactive features include a blood alcohol level calculator, unit counter, ‘thinking drinking record’ of answers to exercises, and email Tips from the DYD Team on cutting down.

**Follow-up data collection**

All participants will be contacted by email three months after baseline data collection to complete follow-up questionnaires online. The emails will contain a hyperlink to the online battery of questionnaires. All participants will receive the same questions at follow-up. Details of the type of data collected at follow-up are provided below under *Outcomes and outcome measures*.

The study will be registered with the UCL data protection officer. The data will be collected and stored in accordance with the Data Protection Act 1998 and will be disposed of in a secure manner. Participants will automatically be assigned a unique identifying number when consenting to take part in the study. All outcome data will be anonymised and identifiable only by the unique ID number. Data will be automatically loaded into a secure, password protected, on-line database, which can only be accessed by the research team. The following measures will be taken to ensure data security:

•The webserver will be protected by a firewall

•Personal data will be encrypted and entered via data entry pages hosted on a Secure Server (https://)

•Email addresses, personal data and research data will be stored in separate, distinct database tables

•Access to database tables will be strictly limited to those actively involved in the project, who will be issued with a username and password

**Outcomes and outcome measures**

The outcomes will reflect the objectives of this feasibility study, which include 1) estimation of study parameters to inform the sample size calculation for a definitive multicentre trial; 2) acceptability and potential risk to employees of participation; 3) engagement with an extensive online alcohol intervention and its suitability for this population.

Outcome measures will include the AUDIT and a questionnaire designed by the research team that explores the acceptability and potential risk of delivering electronic screening and brief intervention to employees in the workplace. The questionnaire will also include questions on whether participants engaged with the online alcohol intervention (detailed below).

*Estimating study parameters*

For organisations that choose to recruit participants via email, rates of eligibility will be determined by the number of employees with a work email address as a percentage of the total workforce at each organisation. These data will be provided by the occupational health leads for each organisation. Occupational health leads will also provide information on the different types of jobs / occupational classifications within the organisation, and which of these require a work email address. This information will highlight any inequalities introduced by restricting inclusion to those employees with a work email address. The number of employees who complete the health screen (and therefore consent to participate in the study) will be reported as a percentage of the total number of employees invited to participate in the study via email, for each organisation. The number of participants scoring five or more on the AUDIT-C will be reported as a percentage of those who have completed the health screen. Finally, the number of participants who complete follow-up questionnaires at three months will be reported as a percentage of those participants completing the health screen, for each organisation.

*Acceptability and potential risk*

The follow-up questionnaire will ask participants about the acceptability of answering questions on their health and receiving feedback online in a workplace setting. There are questions on use of technology, sensitivity of questions, time taken to participate at work and the confidentiality of responses. Participants will be asked if they have any concerns with this approach, including the possible harm to the employee.

*Engagement with online alcohol intervention (Down Your Drink)*

The number of employees from each organisation who access Down Your Drink, i.e. who register with the website, will be reported. The characteristics (i.e. demographics – collected at baseline) of those participants who register with Down Your Drink will be compared descriptively with those participants who did not register. Of those participants that do register with Down Your Drink, the number of logins and number of pages visited per login will be reported; these data will be collected by Google Analytics.

The follow-up questionnaire will include questions about the Down Your Drink website. Firstly, participants will be asked if they accessed the Down Your Drink website. If they did access Down Your Drink, they will be asked what they like about the website, what they dislike and how it could be improved. There will be a list of tick box options to choose from and a free text box for their own comments. Participants who did not access the website will be asked to provide their reasons. Once again they will be presented with a list of tick box options and a free text box for comments. Participants will also be asked if they have accessed any other alcohol-related resources during the three month study period, and if so, to detail what these resources were.

**Analysis**

This feasibility study will aim to recruit (i.e. an employee providing informed consent, completing the health screen and receiving personalised feedback) at least 30 participants from each site to provide reasonable estimates of study parameters ([39](#_ENREF_39)). The analysis will be largely descriptive. Baseline data will be presented as the numbers and percentages of participants who drink above recommended limits, smoke, eat less than 5 portions of fruit and vegetables a day or do less than 150 minutes of moderate or vigorous physical activity a week. These data will also be presented graphically in bar charts. Follow-up AUDIT data will be compared descriptively with baseline AUDIT data. Data from the follow-up questionnaires on the acceptability of the study will be presented as numbers and percentages answering each of the multiple choice questions. These data will also be presented graphically in bar charts to aid interpretation.

Rates of eligibility, recruitment and retention will help determine whether a definitive multicentre trial of eSBI is feasible in these workplace settings, with success criteria detailed in the statistical analysis plan. Responses to questions on the acceptability and potential risk to employees of eSBI in the context of a research study in the workplace will provide more detailed information on whether to proceed to a definitive RCT. The design of a definitive trial will also be informed by the level of engagement with an extensive online alcohol intervention.

**Timelines**

|  | 2012 | | | 2013 | | | | | | | | | | | |
| --- | --- | --- | --- | --- | --- | --- | --- | --- | --- | --- | --- | --- | --- | --- | --- |
|  | Oct | Nov | Dec | Jan | Feb | Mar | Apr | May | Jun | Jul | Aug | Sep | Oct | Nov | Dec |
| Protocol development |  | | | | |  |  |  |  |  |  |  |  |  |  |
| Ethics application and R&D approval |  |  |  |  |  |  |  |  |  |  |  |  |  |  |  |
| Prepare trial website |  |  |  |  |  |  |  |  |  |  |  |  |  |  |  |
| Screening and recruitment |  |  |  |  |  |  |  | |  |  |  |  |  |  |  |
| Three month follow-up period |  |  |  |  |  |  |  | | | |  |  |  |  |  |
| Database cleaning and analysis |  |  |  |  |  |  |  |  |  |  |  | |  |  |  |
| ARUK report and publication preparation |  |  |  |  |  |  |  |  |  |  |  |  | | |  |

**Research team:**

Dr Zarnie Khadjesari

Senior Research Associate, UCL

Dr Elizabeth Murray

Director e-Health Unit, UCL

Dr Dorothy Newbury-Birch

Lecturer in Public Health Research, NCL

Professor Eileen Kaner

Director Institute of Health and Society, NCL

**Project management:**

Don Shenker

Director Alcohol Health Network

**References**

1. Rehm J, Mathers C, Popova S, Thavorncharoensap M, Teerawattananon Y, Patra J. Global burden of disease and injury and economic cost attributable to alcohol use and alcohol-use disorders. Lancet. 2009;373(9682):2223-33. Epub 2009/06/30.

2. The NHS Information Centre. Statistics on alcohol: England, 2012. The Health and Social Care Information Centre, Leeds: 2012.

3. Boniface S, Shelton N. How is alcohol consumption affected if we account for under-reporting? A hypothetical scenario. European journal of public health. 2013. Epub 2013/02/28.

4. Nutt DJ, King LA, Phillips LD. Drug harms in the UK: a multicriteria decision analysis. Lancet. 2010;376(9752):1558-65. Epub 2010/11/03.

5. Prime Minister's Strategy Unit. Alcohol harm reduction strategy for England. London: Cabinet Office, 2004.

6. Anderson P, Baumberg B. Alcohol in Europe: a public health perspective. UK: Institute of Alcohol Studies, 2006.

7. DH / National Treatment Agency for Substance Misuse. Alcohol Misuse Interventions: Guidance on developing a local programme of improvement. London: Department of Health, 2005.

8. Quanbeck A, Lang K, Enami K, Brown RL. A cost-benefit analysis of Wisconsin's screening, brief intervention, and referral to treatment program: adding the employer's perspective. WMJ : official publication of the State Medical Society of Wisconsin. 2010;109(1):9-14. Epub 2010/10/15.

9. NICE public health guidance 24. Alcohol-use disorders: preventing the development of hazardous and harmful drinking. London: National Institute for Health and Clinical Excellence, 2010.

10. Kaner EF, Beyer F, Dickinson HO, Pienaar E, Campbell F, Schlesinger C, et al. Effectiveness of brief alcohol interventions in primary care populations. Cochrane Database Syst Rev. 2007(2):CD004148. Epub 2007/04/20.

11. Moyer A, Finney JW, Swearingen CE, Vergun P. Brief interventions for alcohol problems: a meta-analytic review of controlled investigations in treatment-seeking and non-treatment-seeking populations. Addiction (Abingdon, England). 2002;97(3):279-92. Epub 2002/04/20.

12. Ballesteros J, Duffy JC, Querejeta I, Arino J, Gonzalez-Pinto A. Efficacy of brief interventions for hazardous drinkers in primary care: systematic review and meta-analyses. Alcoholism: Clinical and Experimental Research. 2004;28(4):608-18. Epub 2004/04/22.

13. Bien TH, Miller WR, Tonigan JS. Brief interventions for alcohol problems: a review. Addiction (Abingdon, England). 1993;88(3):315-35. Epub 1993/03/01.

14. Poikolainen K. Effectiveness of brief interventions to reduce alcohol intake in primary health care populations: a meta-analysis. Preventive medicine. 1999;28(5):503-9. Epub 1999/05/18.

15. Whitlock EP, Polen MR, Green CA, Orleans T, Klein J, Force USPST. Behavioral counseling interventions in primary care to reduce risky/harmful alcohol use by adults: a summary of the evidence for the U.S. Preventive Services Task Force. Annals of internal medicine. 2004;140(7):557-68. Epub 2004/04/08.

16. Heather N. Can screening and brief intervention lead to population-level reductions in alcohol-related harm? Addiction Science & Clinical Practice. 2012;7(15):1-14.

17. Hutchings D, Cassidy P, Dallolio E, Pearson P, Heather N, Kaner E. Implementing screening and brief alcohol interventions in primary care: views from both sides of the consultation. Primary Health Care Research & Development. 2006;7(03):221-9.

18. Johnson M, Jackson R, Guillaume L, Meier P, Goyder E. Barriers and facilitators to implementing screening and brief intervention for alcohol misuse: a systematic review of qualitative evidence. Journal of Public Health. 2011;33(3):412-21. Epub 2010/12/21.

19. McAvoy BR, Donovan RJ, Jalleh G, Saunders JB, Wutzke SE, Lee N, et al. General Practitioners, Prevention and Alcohol - a powerful cocktail? Facilitators and inhibitors of practising preventive medicine in general and early intervention for alcohol in particular: a 12-nation key informant and general practitioner study. Drugs: Education, Prevention, and Policy. 2001;8(2):103-17.

20. Rapley T, May C, Frances Kaner E. Still a difficult business? Negotiating alcohol-related problems in general practice consultations. Social Science and Medicine. 2006;63(9):2418-28. Epub 2006/07/04.

21. Wutzke SE, Donovan RJ, Gomel MK. Enhancing the delivery of brief interventions for hazardous alcohol use in the general practice setting: a role for both general practitioners and medical receptionists. Health Promotion Journal of Australia. 1998;8(2):1036-73.

22. Watson H, Godfrey C, McFadyen A, McArthur K, Stevenson M. Reducing Alcohol-Related Harm in the Workplace. A Feasibility Study of Screening and Brief Interventions for Hazardous Drinkers. Glasgow: Caledonian Nursing and Midwifery Research Centre, Glasgow Caledonian University, 2009.

23. Webb G, Shakeshaft A, Sanson-Fisher R, Havard A. A systematic review of work-place interventions for alcohol-related problems. Addiction (Abingdon, England). 2009;104(3):365-77. Epub 2009/02/12.

24. Burton J. World Health Organization Healthy Workplace Framework and Model: Background and supporting literature and practices. Geneva: 2010.

25. NICE public health guidance 13. Promoting physical activity in the workplace. Manchester: National Institute for Health and Clinical Excellence, 2008.

26. Del Boca FK, Darkes J. The validity of self-reports of alcohol consumption: state of the science and challenges for research. Addiction (Abingdon, England). 2003;98 Suppl 2:1-12. Epub 2004/02/27.

27. Khadjesari Z, Murray E, Hewitt C, Hartley S, Godfrey C. Can stand-alone computer-based interventions reduce alcohol consumption? A systematic review. Addiction (Abingdon, England). 2011;106(2):267-82. Epub 2010/11/19.

28. Rooke S, Thorsteinsson E, Karpin A, Copeland J, Allsop D. Computer-delivered interventions for alcohol and tobacco use: a meta-analysis. Addiction (Abingdon, England). 2010;105(8):1381-90. Epub 2010/06/10.

29. White A, Kavanagh D, Stallman H, Klein B, Kay-Lambkin F, Proudfoot J, et al. Online alcohol interventions: a systematic review. Journal of medical Internet research. 2010;12(5):e62. Epub 2010/12/21.

30. Riper H, Spek V, Boon B, Conijn B, Kramer J, Martin-Abello K, et al. Effectiveness of E-self-help interventions for curbing adult problem drinking: a meta-analysis. Journal of medical Internet research. 2011;13(2):e42. Epub 2011/07/02.

31. Carey KB, Scott-Sheldon LA, Elliott JC, Bolles JR, Carey MP. Computer-delivered interventions to reduce college student drinking: a meta-analysis. Addiction (Abingdon, England). 2009;104(11):1807-19. Epub 2009/09/12.

32. Bewick BM, Trusler K, Barkham M, Hill AJ, Cahill J, Mulhern B. The effectiveness of web-based interventions designed to decrease alcohol consumption--a systematic review. Preventive medicine. 2008;47(1):17-26. Epub 2008/02/28.

33. Elliott JC, Carey KB, Bolles JR. Computer-based interventions for college drinking: a qualitative review. Addictive behaviors. 2008;33(8):994-1005. Epub 2008/06/10.

34. Doumas DM, Hannah E. Preventing high-risk drinking in youth in the workplace: a web-based normative feedback program. Journal of substance abuse treatment. 2008;34(3):263-71. Epub 2007/06/30.

35. Matano RA, Koopman C, Wanat SF, Winzelberg AJ, Whitsell SD, Westrup D, et al. A pilot study of an interactive web site in the workplace for reducing alcohol consumption. Journal of substance abuse treatment. 2007;32(1):71-80. Epub 2006/12/19.

36. Murray E, Khadjesari Z, Linke S, Hunter R, Freemantle N. Health on the web: randomised trial of work-based online screening and brief intervention for hazardous and harmful drinking. BMC public health. 2013;13(1):505. Epub 2013/05/28.

37. Bush K, Kivlahan DR, McDonell MB, Fihn SD, Bradley KA. The AUDIT alcohol consumption questions (AUDIT-C): an effective brief screening test for problem drinking. Ambulatory Care Quality Improvement Project (ACQUIP). Alcohol Use Disorders Identification Test. Archives of internal medicine. 1998;158(16):1789-95. Epub 1998/09/17.

38. Babor TF, Higgins-Biddle JC, Saunders JB, Monteiro MG. AUDIT - The Alcohol Use Disorders Identification Test: Guidelines for Use in Primary Care. Geneva: World Health Organization, 2001.

39. Lancaster GA, Dodd S, Williamson PR. Design and analysis of pilot studies: recommendations for good practice. Journal of evaluation in clinical practice. 2004;10(2):307-12. Epub 2004/06/11.
